# Land Subsidence Related to Coal Mining in China Revealed by L-Band InSAR Analysis

**DOI:** 10.3390/ijerph17041170

**Published:** 2020-02-12

**Authors:** Liping Zheng, Lin Zhu, Wei Wang, Lin Guo, Beibei Chen

**Affiliations:** 1 Laboratory Cultivation Base of Environment Process and Digital Simulation, College of Resource Environment and Tourism, Capital Normal University, Beijing 100048, China; 2170902095@cnu.edu.cn (L.Z.); 6183@cnu.edu.cn (B.C.); 2 Tianjin Center of China Geological Survey, Tianjin 300170, China; wangwei_wangwei@126.com

**Keywords:** land subsidence, InSAR, potential risk region, coal mining, Ordos

## Abstract

Geological disasters, including ground deformation, fractures and collapse, are serious problems in coal mining regions, which have threatened the sustainable development for local industry. The Ordos Basin is most known for its abundant coal resources. Over-mining the underground coal resources had induced land deformation. Detecting the evolution of the land deformation features and identifying the potential risk are important for decision-makers to prevent geological disasters. We analyzed land subsidence induced by coal mining in a 200 ${\mathrm{km}}^{2}$ area in the Ordos Basin for the time period 2006–2015. ALOS-1 PALSAR images from December 2006 to January 2011 and ALOS-2 PALSAR-2 images from December 2014 to July 2015, optical remotely sensed images and coal mining information were collected. The small baseline subset interferometric synthetic aperture radar (SBAS-InSAR) method and differential interferometric synthetic aperture radar (D-InSAR) method, GIS and statistical analysis were adopted. Results show that the maximum subsidence rate and cumulative subsidence along the line of sight (LOS) were −65 mm/year and −246 mm, respectively, from December 2006 to January 2011. The maximum cumulative subsidence was −226 mm from December 2014 to July 2015. The new boundary of the mining goafs from 2014 to 2015 and the most dangerous risk region were mapped. Moreover, the effect of large-scale mining coal, with the production volume exceeds 1.2 million tons per year, with the operation time more than 20 years on land subsidence was found greater than small and medium-scale coal mines and reached −59 mm/year. The recently established small-sized and medium-sized coal mines show high land subsidence. This study will contribute to better understand the land subsidence process in mining region and provide scientific support for government to prevent land subsidence.

## 1. Introduction

Coal is the most important primary energy source, accounting for more than 70% of total primary energy consumption in China [1]. According to the National Bureau of Statistics, China’s coal production in 2016 was 3.41 billion tons, accounting for 45.7% of the world’s total output. Ordos, located in Inner Mongolia, China, is one of the important locations for super coal mines. In 2018, the production of coal in Ordos reached 616 million tons. Along with a large number of coal mining regions in Ordos, ground fissures, ground collapse and land subsidence have undermined the land surface ecosystems [2]. In Yongcheng City, located in Henan Province, another region in China with abundant coal resources, land subsidence and ground collapse are two geological phenomena that have occurred [3]. In that mining area, ground collapse is easily identified due to land deformation of up to a few meters during the active coal mining period. Land subsidence is a slow change process compared with land collapse, which can develop into ground collapses, so it is necessary to understand the land subsidence process in mining areas.

In order to monitor land subsidence in coal mining areas, conventional monitoring methods with high accuracies have been applied, including geodesy, precise leveling, close range photogrammetry and GPS [4]. However, these methods are limited by heavy workload and high cost. The InSAR techniques have significant advantages over other conventional monitoring methods, and are widely used to monitor land subsidence [5,6,7,8,9]. The advantages include wide spatial coverage, good working capabilities in all climatic conditions, high measurement precision and no need for ground instrumentation [10,11]. The first application of the InSAR technique was to monitor an earthquake in Landers (California, USA) [12]. The results successfully provided a coseismic deformation map comparable to the 92 GPS measurements available. Then, Differential Interferometric Synthetic Aperture Radar (D-InSAR), as an extension to interferometric synthetic aperture radar, has been developed and give the measurement of land subsidence caused by glacial kinematics [13,14], volcanic-tectonic deformation [15,16] and other anthropogenic activities, such as underground mining and groundwater extraction [4]. The D-InSAR technique can theoretically detect surface changes with centimeter [11,17,18]. It is suitable for short-term measurements, but the application of D-InSAR is seriously affected by atmospheric delay noise and spatio-temporal incoherence, which are caused by atmospheric fluctuations, different satellite observation positions, and long image-acquisition intervals, respectively [6]. Permanent scatterers InSAR (PSI), small baseline subset InSAR and other time series InSAR techniques have the advantage of overcoming the problems of traditional InSAR techniques [19,20,21,22,23,24]. All of these time-series InSAR techniques have the capability to extract long-term surface deformation and have been successfully applied in monitoring land subsidence due to underground resources exploitation and urban exploitation [25,26,27].

Moreover, as many SAR satellite plans are developing, there are many SAR sensor images with different bands, such as X-band, L-band and C-band. The relatively long wavelength of L-band SAR images makes them able to detect large deformations. ALOS-PALSAR images were generally used by researchers to monitor land subsidence in coal mines area [28,29]. Based on the land subsidence information, the main factors of land subsidence over coal mining areas were analyzed. For example, the relationships between mining exploitation, groundwater storage change and surface deformation were studied [24,30]. The results show that land deformation is mainly due to the underground mine exploitation and residual subsidence in the goafs. In India, the displacement-affected area was found generally larger than the extracted mining area [31]. Moreover, a combined analysis of subsidence magnitudes and horizontal gradient can indicate the higher vulnerability areas to surface faulting. Large subsidence gradients found to coincide with prominent surface faults in central Mexico [32]. Therefore, studies on the land subsidence in coal mining area mainly focuses on the monitoring methods and the subsidence mechanisms. 

In Ordos, as the main coal resources region, land surface subsidence due to groundwater extraction and underground mining activities was studied [33,34]. There are few studies based on coal mining information to obtain the spatial-temporal characteristics of coal mining areas. The aim of this study is to detect the features of land subsidence in Ordos by InSAR techniques in 2006–2015 and to deduce the risk region around the boundary of mining goafs. During the process, the effects of coal mining scales on land subsidence were analyzed by spatial analysis and statistical analysis. The results of this study will provide scientific support for decision-maker to manage the geological environmental problems in mines.

## 2. Study Area

**Figure 1:**
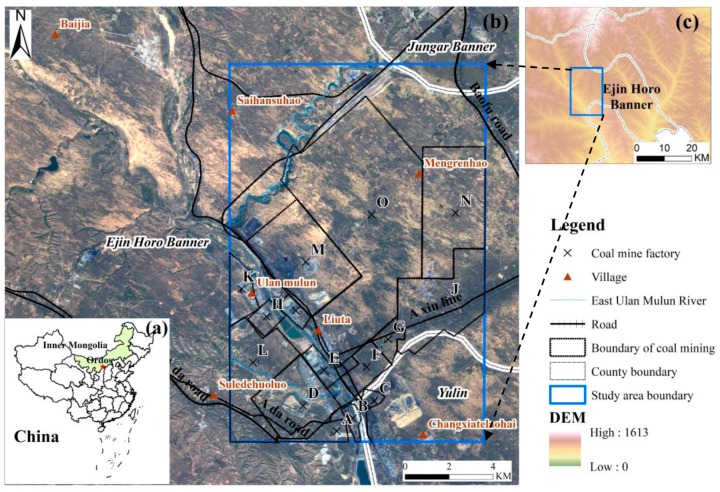
(**a**) Study area position on the map of China with the location of Ordos marked with a red star; (**b**) the geographic location of the study area is indicated by the blue polygon, which is superimposed on the SPOT-6 image obtained in 2015; (**c**) the elevation of study area in Ejin Horo Banner, Ordos, Inner Mongolia Autonomous Region.

The south-eastern of Ordos, Inner Mongolia, China (Figure 1a,b) is chosen as the study area. It is a region known for its abundance of coal resources with an area of 200 ${\mathrm{km}}^{2}$. The elevation ranges from 1000 m and 1500 m. The topography is high in the south and low in the northern part of the Inner Mongolian Plateau. The area belongs to the arid-semiarid regions with a mean annual precipitation of 351 mm from 1984 to 2015, which is generally concentrated from July to September in the survey data collected from coal mine factories. The East Ulan Mulun River runs across the study area (Figure 1b), and villages are distributed along this river. Xin Road and Da Road are the two main transportation routes, which deliver the coal resources to other places. 

Moreover, the study area is a transition zone between crop areas and nomadic areas, where desertification has occurred with sparse vegetation cover. The material of 75% of the deposits is Quaternary aeolian sand and loess. Fifteen coal mines were built in this study area, with a mining area of about 30 ${\mathrm{km}}^{2}$ until 2012. This mining activity had induced ground collapse and ground fissures. The survey data which was collected from coal mine factories shows that about 14 ${\mathrm{km}}^{2}$ ground collapse and 1.64 ${\mathrm{km}}^{2}$ of ground fissures can be found there.

## 3. Datasets and Methodology

### 3.1. Datasets

To detect the features of land subsidence and the potential risk region in coal mining area, SAR images including ALOS-1 PALSAR and ALOS-2 PALSAR-2 acquired by the Japanese Space Agency (JAXA) and the Japanese Ministry of Economy, Trade and Industry (METI), optical images including SPOT-6 and Google Earth, and coal mining information were gathered.

#### 3.1.1. SAR Remotely Sensed Images

The Advanced Land Observing Satellite PALSAR sensor with a 10 m spatial resolution, which has a long wavelength of L band is known to be more advantageous than other shorter wavelength microwave bands in terms of interferometric coherence [35]. In this article, a total of 22 images were chosen to detect the regional land subsidence. Detailed information on the satellite sensors is presented in Table 1.

#### 3.1.2. Optical Remotely Sensed Images

SPOT-6 image with the spatial resolution of 1.5 m in October 2015 and long-term series Google Earth data during the period from 2006 to 2011 were collected to identify changes in the land surface and compare the output of SAR images.

#### 3.1.3. Coal Mining Information

There are total of 15 factories named A to O in the 200 ${\mathrm{km}}^{2}$ study area. They are classified into three scales according to the mining capacity. The classification criteria are based on annual output of $4.5\times 10^{5}$ tons or less, $4.5\times 10^{5}$ to 1.2 million tons and more than 1.2 million tons [36]. All the factories were established at different times, with the earliest established in 1965, and the latest completed in 2011. The coal mining information collected from coal mine factories in 2012 is given in Table 2.

### 3.2. Methodology

**Figure 2:**
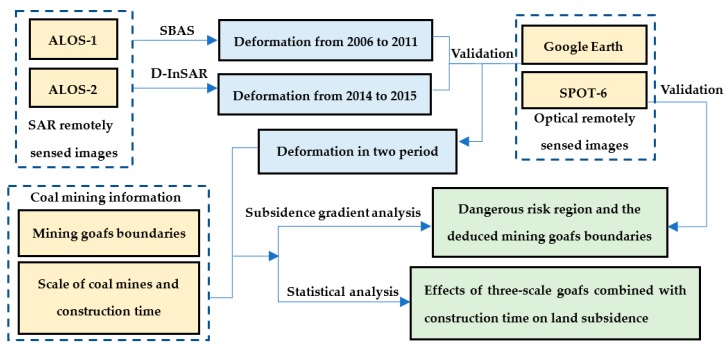
Flowchart of methodology to monitor land deformation over the coal mining area in the study area. Pink represents input data, Blue represents the InSAR output data, Green represents the analysis output data.

To detect the evolution of land subsidence over the two period, from December 2006 to January 2011 and December 2014 to July 2015, InSAR techniques and GIS analysis were integrated. The processing flowchart is presented in Figure 2. Considering the high vegetation coverage, SBAS-InSAR and DInSAR techniques were adopted to obtain surface displacements from December 2006 to January 2011 and December 2014 to July 2015, respectively.

#### 3.2.1. Detection Land Subsidence by Means of Small Baseline InSAR Technique from 2006 to 2011

The small baseline subset interferometric synthetic aperture radar (SBAS-InSAR) technique was proposed by Berardino et al. and implemented to derive time series of ground deformation with millimetric accuracy [20]. In this technique, temporal sampling of deformation monitoring can be increased by combining interferograms with small baselines and the geometric and temporal decorrelation effects can be reduced by small baselines. After that, different subsets of interference pairs which are separated by large baselines were created [37]. This technique also shows applicability in non-urban (rural) areas which are characterized by vegetated or low reflectivity homogenous regions [38]. 

In the data processing, Gamma software was used to interpret land subsidence information. The maximum perpendicular baseline difference and maximum temporal difference were 2500 m and 300 days, respectively. The multi-look factors were set 3 to 7 to improve the noise ratio signal. And 47 pairs of interferograms were generated using the multiple-master image method. The external SRTM DEM of 30 m was then used to remove the effect of the topographic phase. And the atmospheric delays phase could be mitigated by using spatio-temporal filtering. In the end, a total of $2.88\times 10^{5}$ highly coherent points were extracted with temporal coherence greater than 0.7 from 2006 to 2011.

#### 3.2.2. Detection Land Subsidence by Means of DInSAR Technique from 2014 to 2015

**Figure 3:**
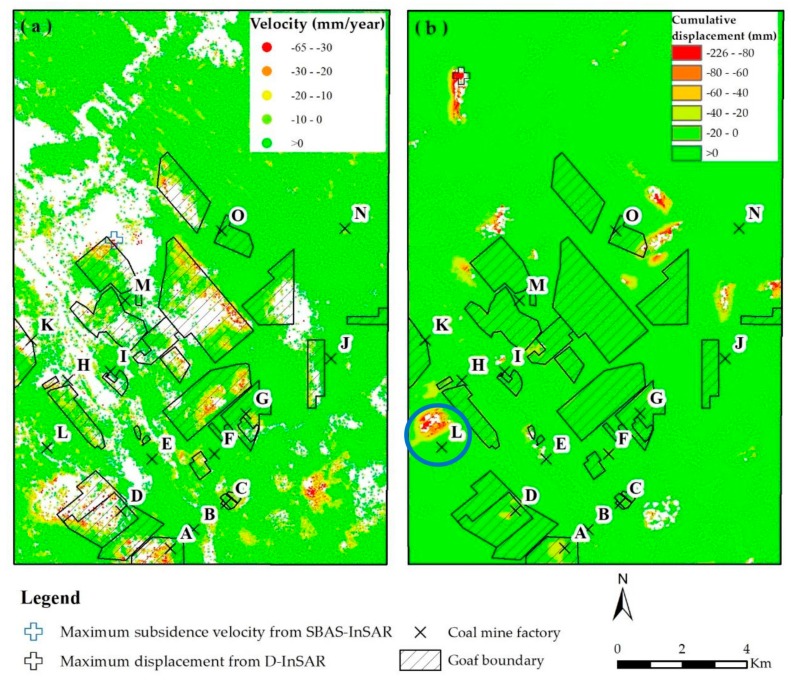
(**a**) Distribution of land subsidence velocity derived with SBAS-InSAR technique from December 2006 to January 2011; (**b**) distribution of the cumulative displacement through DInSAR technique from December 2014 to July 2015.

**Figure 4:**
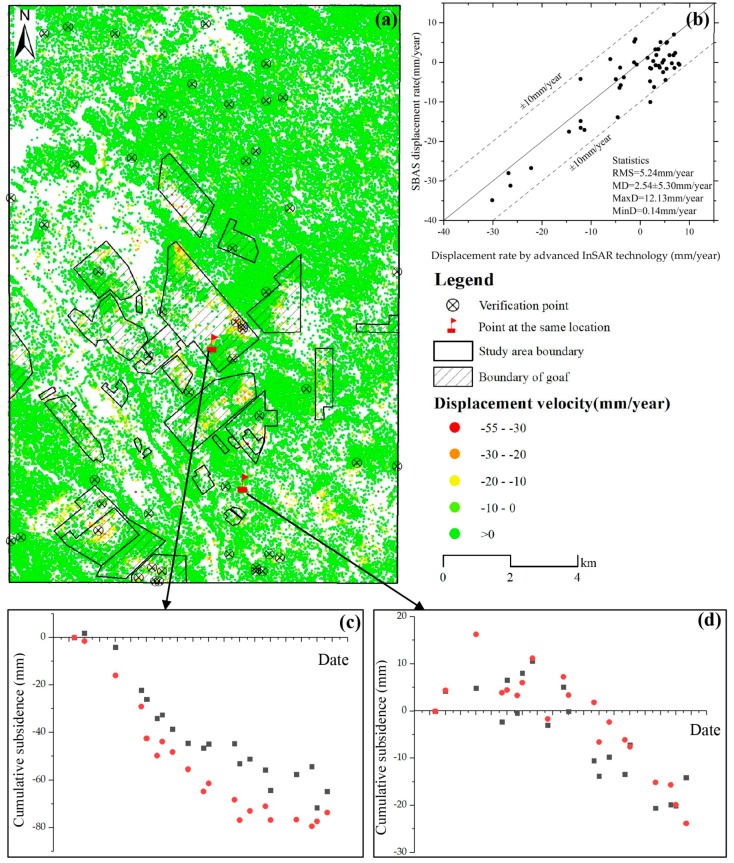
(**a**) Time-series InSAR result from 2006 to 2011 combining with distributed scatterers and permanent scatterers; (**b**) comparison of land subsidence obtained by two InSAR techniques; (**c**,**d**) cumulative displacement at the same location obtained by two approaches. Red dot represents SBAS-InSAR result and the black square represents the InSAR result which combined with distributed scatterers and permanent scatterers.

**Figure 5:**
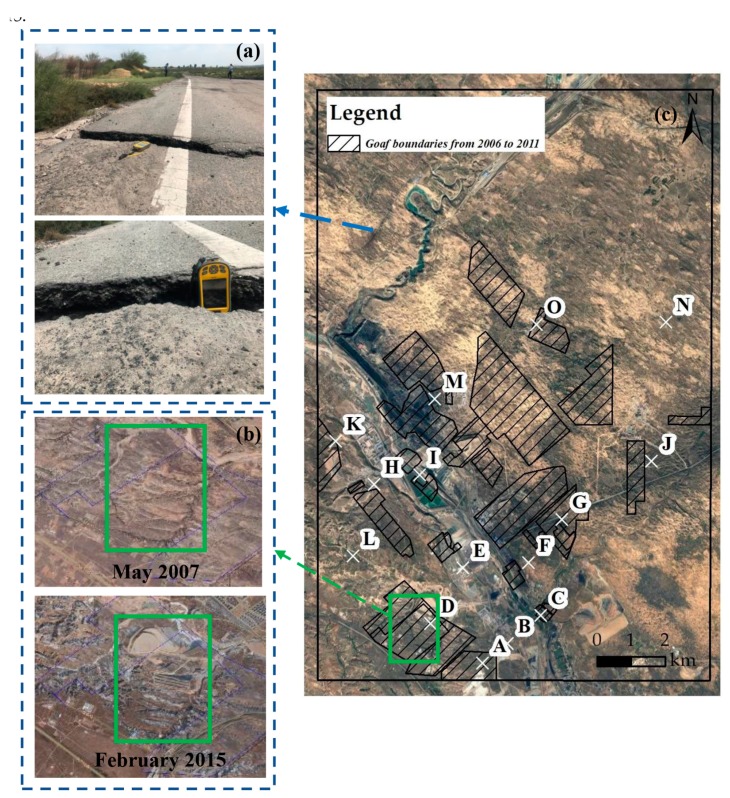
(**a**) Ground fissures images obtained during a field survey; (**b**) historical images of Google Earth within the boundaries of the coal mining goafs in 2007 and 2015; (**c**) location of the coal mining goafs in the mining area.

In this article, the D-InSAR technique was applied to two ALOS-PALSAR 2 images from December 2014 to July 2015 and the image co-registration is based on the master image, which the perpendicular baseline difference and temporal baseline difference were 147m and 224 days, respectively. After that, an interferogram was generated and the interferometric phase was subtracted from the simulated topographic phase, which was formed by an external SRTM DEM of 30 m [39], to remove the effect of the topographic phase. Moreover, the minimum cost flow (MCF) algorithm was the crucial step to integral deformation. The results derived by InSAR techniques are shown in Figure 3. And the validation of the SAR outcomes is described in Section 4.2 (Figure 4 and Figure 5).

#### 3.2.3. The Subsidence Gradient Calculation and Analysis Method

The gradient of land subsidence is used to identify the risk region of subsidence in two periods. It computed as the difference in subsidence between adjacent cells, divided by their horizontal and vertical distance [32,40]. The equations of subsidence gradient (in degrees) are expressed as follows:

(1) $$\mathrm{Gradient}\;=\mathrm{Arctan}\sqrt{{\left(\frac{d_{z}}{d_{x}}\right)}^{2}+{\left(\frac{d_{z}}{d_{y}}\right)}^{2}}*\;57.29578\;\mathrm{degrees}$$

where $d_{x}$ and $d_{y}$ are the distance from X axis and Y axis, respectively. $d_{z}$ is the differential subsidence between measurement pixels.

**Figure 6:**
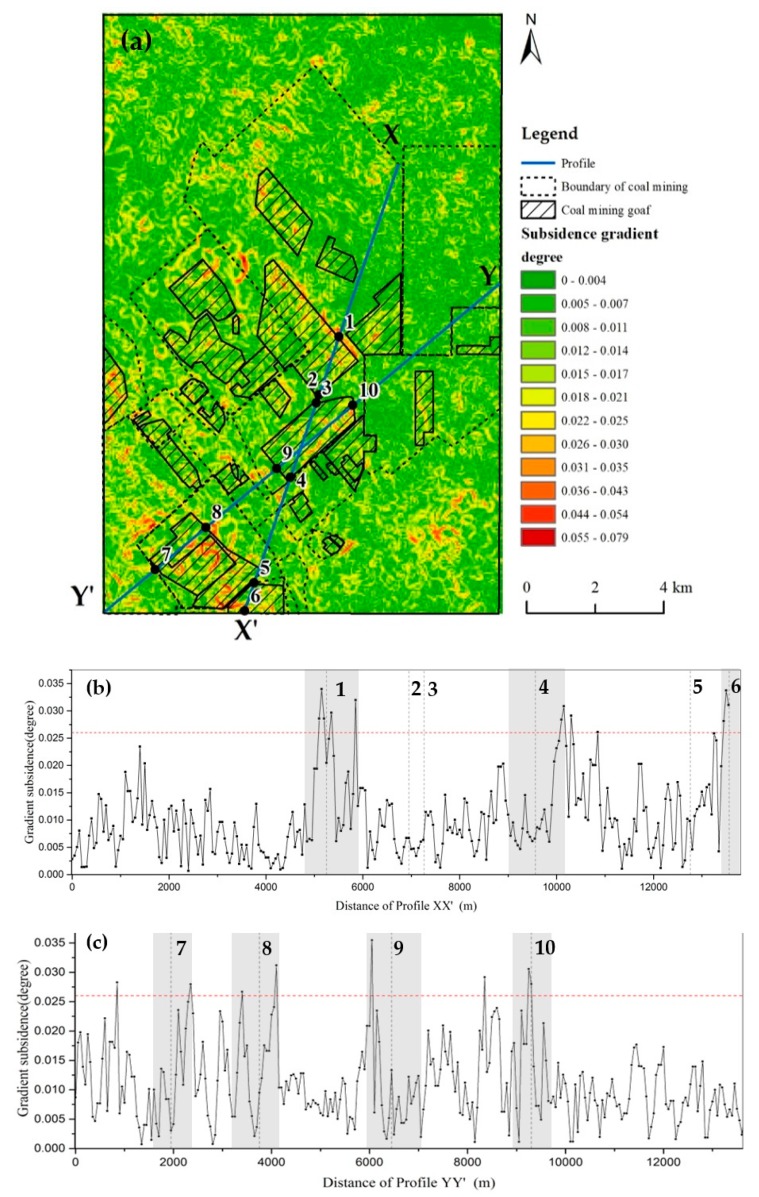
(**a**) Distribution of subsidence gradient during the period from 2006 to 2011; (**b**,**c**) the subsidence gradient along the lines of XX’ and YY’; the index number points were the intersection between the boundary of goafs and profile lines; the gray shaded area is the zone where the subsidence gradient exceeds 0.026 degree around the junction of the goaf boundary with two profile lines.

In this article, cumulative displacement raster datasets with 50 m resolution from December 2006 to January 2011 were created from measurement pixels by Kriging interpolation to obtain spatially continuous land subsidence distribution. Then, the subsidence gradient map calculated by Equation (1) is given in Figure 6a. The natural breaks (Jenks) method is used to identify the class breaks which can best group similar values and maximize the differences between classes [41]. After defining the major classes, the subsidence gradient within buffer zone of a certain width around the boundary of the goafs were analyzed to deduce risk region around the boundary of mining goafs.

## 4. Results

### 4.1. Distribution of Land Subsidence in Coal Mining Area

The maximum subsidence rate and cumulative subsidence in the LOS direction were −65 mm/year and −246 mm from December 2006 to January 2011, respectively. These two values are located near the M coal mining goaf (marked with blue cross in Figure 3). 

Moreover, several strip-shaped subsidence zones (with red points) with the subsidence rate exceeded −20 mm/year, were found formed along the coal mining goafs in the middle of the study area, except for the subsidence area in the southeast. This indicates that there is a preferential spatial correlation between land subsidence and the mining area. For the subsidence area in the southeast of the study area, there may be illegal mining occurring.

From December 2014 to July 2015, the maximum cumulative subsidence along the LOS was −226 mm, which was outside the coal mining boundary, located in the northwestern part of the study area. The reasons why the subsidence area is outside the coal mining goafs may be a change in the location of the mining area. Moreover, the area with the cumulative subsidence zones exceeding -50 mm (marked with orange and red color in Figure 3b) was more than 0.07 ${\mathrm{km}}^{2}$. The main subsidence zones were located in the L coal mine in the southwestern of the study area (marked with blue circle in Figure 3b), with an area of 0.52 ${\mathrm{km}}^{2}$. These subsidence zones were distributed outside of the original investigated coal mining goafs, which may indicate the new mining area or human activity in these regions. The uplift value may be caused by the residual atmospheric error phase or the soil or tailing stacking in mining areas.

### 4.2. Validation of the SAR Outcomes

Due to the lack of levelling data in the study area, cross-validation method was used to test the accuracy of the land subsidence results from 2006 to 2011. The subsidence results by using advanced InSAR technique, which combined distributed scatterer (DS) and permanent scatterer (PS) [42,43], were shown in Figure 4a. This technique presented an approach to retrieve surface deformation over nonurban areas, which can significantly increase the spatial density of measurement points and revealed the potential of the monitoring approach in suburban areas [42,43]. In this article, the results from both the advanced InSAR technique and SBAS-InSAR technique were interpreted relative to the same reference point.

The results (Figure 4a) of the advanced InSAR technique revealed that the maximum LOS subsidence rate and cumulative LOS subsidence in the coal mining area were −55 mm/year and -206 mm, respectively. And the maximum LOS subsidence rate of SBAS-InSAR technique results was −65 mm/year, of which only four points had subsidence rate exceeding −55 mm/year. Fifty points were randomly chosen to validate the subsidence results from SBAS-InSAR and advanced InSAR technique. The correlation coefficient between the two different techniques was 0.89 and the root mean square was 5.24 mm/year, which can reflect the reliability of the SBAS-InSAR derived land subsidence. Two points at the same location obtained with the two approaches are selected to compare the cumulative subsidence (Figure 4c,d) from December 2006 to January 2011. It can be found that the two cumulative subsidence trends are consistent.

Google Earth’s historical images were used to explain the existence of coherence loss, which is shown as blank spaces in Figure 3. Google Earth images obtained between 2007 and 2015 are given in Figure 5b. The ground surface undergone drastic changes, which results in loss of coherence and thus, it is hard to extract the measurement points (marked with green rectangles in Figure 5). The ground fissures images obtained by field survey in July 2017 located in the northwestern part of the study area and are shown in Figure 5a. As the location is near the subsidence zone in Figure 3b, it also indicates the reliability of D-InSAR technique interpretation from December 2014 to July 2015.

## 5. Discussion

### 5.1. Dangerous Risk Region around the Boundary of Mining Goafs

The gradient of subsidence is divided into 12 classes by the natural breaks (Jenks) method. And the subsidence gradient was generally found high at the edge of subsidence funnel with the value larger than 0.026 degree in Figure 6a. Two lines of XX’ and YY’ crossing the coal mining area were used to illustrate the spatial characteristics of the land subsidence gradient (Figure 6b,c). There is a large subsidence gradient (highlighted by grey polygon in Figure 6b,c) within a range of the boundary of the coal mining goafs. The red horizontal line in Figure 6b, c refers to a subsidence gradient of 0.026 degree. There are seven points of intersection between the boundary of coal mining goafs and the profile lines with the subsidence gradient exceeding 0.026 degree. These points are Point 1, Point 4 and Point 6 along XX’ profile line and Point 7, Point 8, Point 9 and Point 10 along YY’ profile line. The large gradient indicated the severely uneven displacement, which facilitated the occurrence of surface fissures and ground collapse.

**Figure 7:**
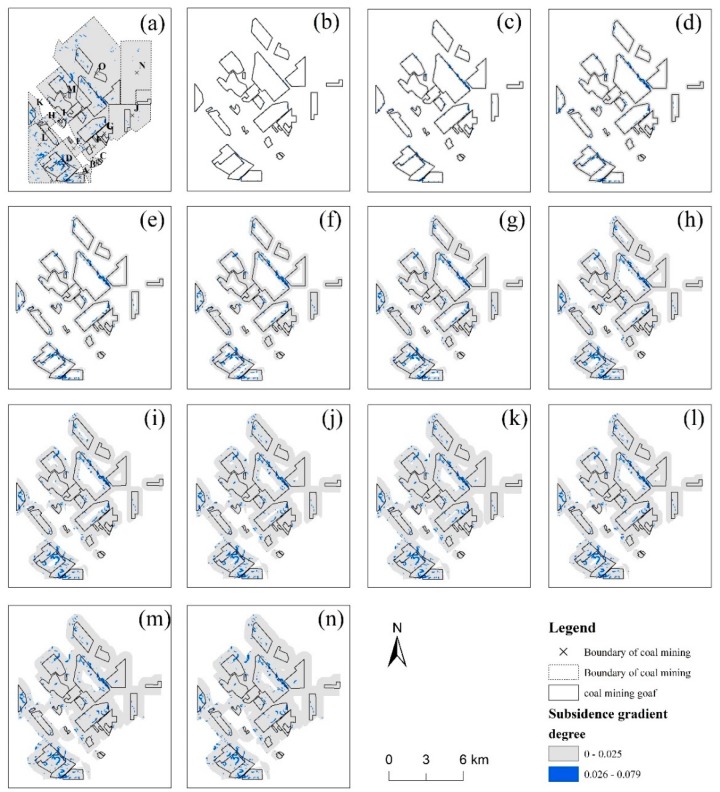
(**a**) Subsidence gradient in the entire coal mining area; (**b**–**n**) subsidence gradient map with buffers along the goafs boundary ranging from 50 m to 650 m with an interval of 50 m; the blue pixels represent the high subsidence gradient.

**Figure 8:**
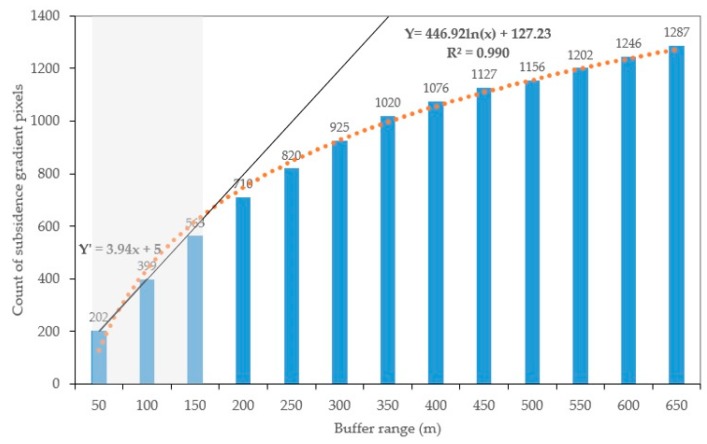
Statistical results of the subsidence gradient in the coal mining area from 2006 to 2011. The blue columns of the histogram refer to the count of subsidence gradient pixels in the different buffer zones.

The high subsidence gradient within the buffer zones of the goaf boundaries ranging from 50 to 650 m with an interval of 50 m (b to n) were calculated and were shown in Figure 7 and Figure 8. A logarithmic fitting analysis of the count of subsidence gradient pixels in different buffer zones indicated that the increase of the buffer zones reduces the growth rate of the subsidence gradient pixels count. Also, a linear fitting analysis of the count of subsidence gradient pixels in 50 m and 100 m buffer zones showed that the results were beyond the trend of logarithmic fitting in the 150 m buffer zones.

Furthermore, to find the drastic change range, the change rates of high subsidence gradient areas between two adjacent buffer zones were calculated and were shown in Table 3. It can be seen that within the buffer zones of 50 m to 150 m around the goaf areas, the maximum change rates of areas with subsidence gradient values greater than three which are higher than those of other buffer zones. The value of the change rate dropped from 3.28 to 0.82 within the range of 100 to 650 m buffer zones. Therefore, the range of 50 m to 150 m was regarded as the dangerous boundary around mining goafs.

**Figure 9:**
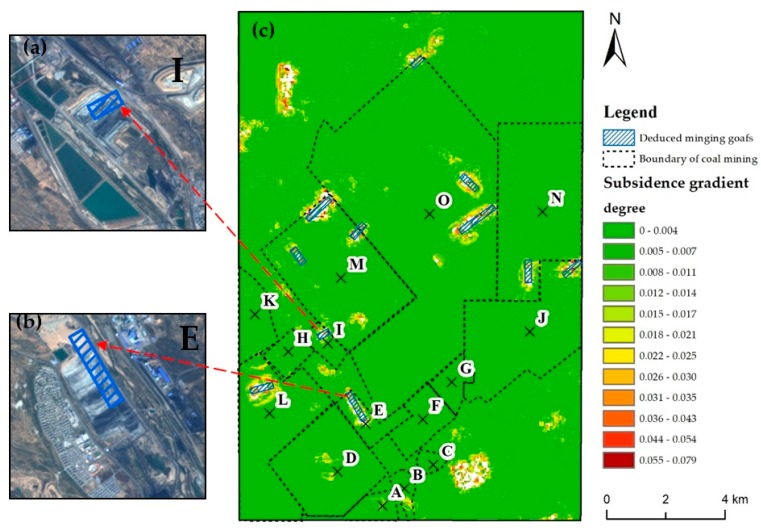
(**a**,**b**) The corresponding SPOT-6 images with respect to the deduced goaf boundaries in E and I coal mines; (**c**) the deduced boundaries of mining goafs from 2014 to 2015 (blue lines) superimposed on the subsidence gradient map.

According to the subsidence gradient map and the dangerous risk region, the boundaries of coal mining goafs from December 2014 to July 2015 could be deduced. The total of 11 goaf boundaries deduced were marked with blue lines in Figure 9c. Moreover, in the E and I mining areas, mining activities were found in the deduced coal mining goafs in superimposed SPOT-6 optical images from October 2015. 

### 5.2. The Effects of Mining Scales in the Coal Mining Goafs on Land Subsidence

Combined with the land subsidence results from 2006 to 2011, a total of 5741 measurement pixels in three scales of coal mine goafs was statistically analyzed. The results are shown in Table 4.

**Figure 10:**
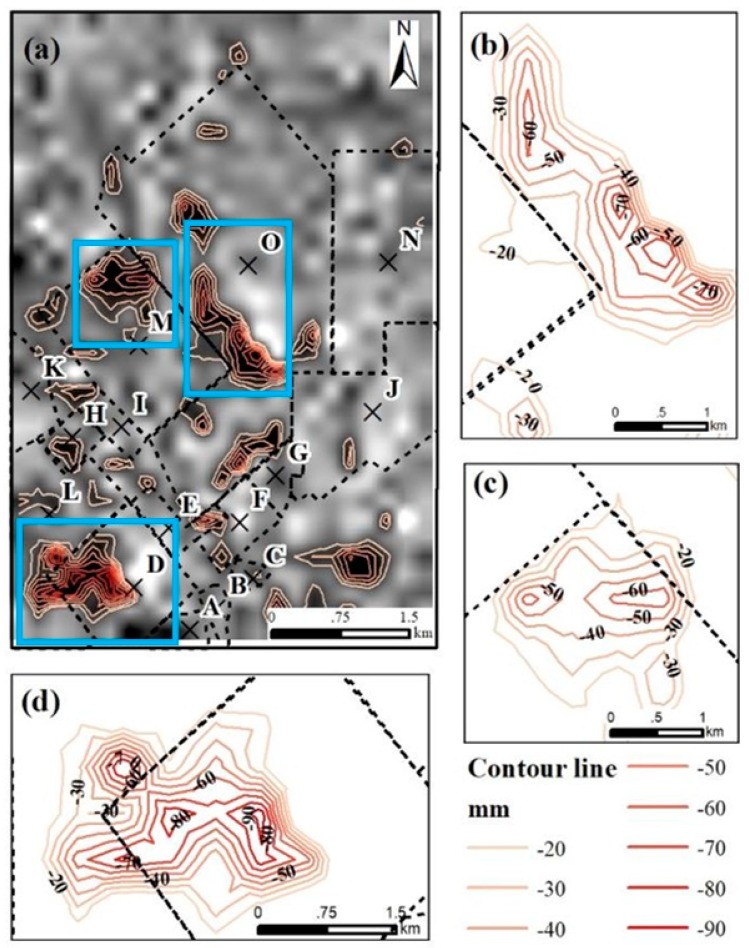
(**a**) Map of the deformation contours obtained from the cumulative displacement from December 2006 to January 2011; (**b**–**d**) the local deformation contours in the O, M and D coal mines.

The proportion of pixels with subsidence rates of more than −10 mm/year for small-scale mining goafs was approximately equal to that of medium-scale mines. The maximum subsidence rate of small coal mines goafs was −26.69 mm/year, which was slightly greater than that of the medium-scale mines. For the large coal mines goafs, the proportion of pixels with subsidence rate exceeding -10 mm/year was about 18, which was obviously larger than that in small and medium-scale coal mines goafs. The maximum subsidence rate was about −59 mm/year and located in D coal mine factory. Moreover, the deformation contours from 2006 to 2011 (Figure 10) showed that there were three severe subsidence areas, which distributed in the northwest and southwest part in this study area. The area of three subsidence regions were 4.14 ${\mathrm{km}}^{2}$ (Figure 10b), 3.19 ${\mathrm{km}}^{2}$ (Figure 10c) and 4.89 ${\mathrm{km}}^{2}$ (Figure 10d), respectively. These regions were all located in the large-scale coal mines area named O, M and D coal mine factory. The maximum deformation contour was −90 mm which located in the large-scale coal mines named D. 

Due to the construction time of O, M and D coal mine factories, the operating time of these coal mines until January 2011 is 22 years. It can be found that during 2006–2011, large coal mine factories which operating more than 20 years are more likely to induce land subsidence in this region. That is the large-scale mines with large mining capacity output of 1.2 million tons and operating more than 20 years are likely to induce land subsidence. 

**Figure 11:**
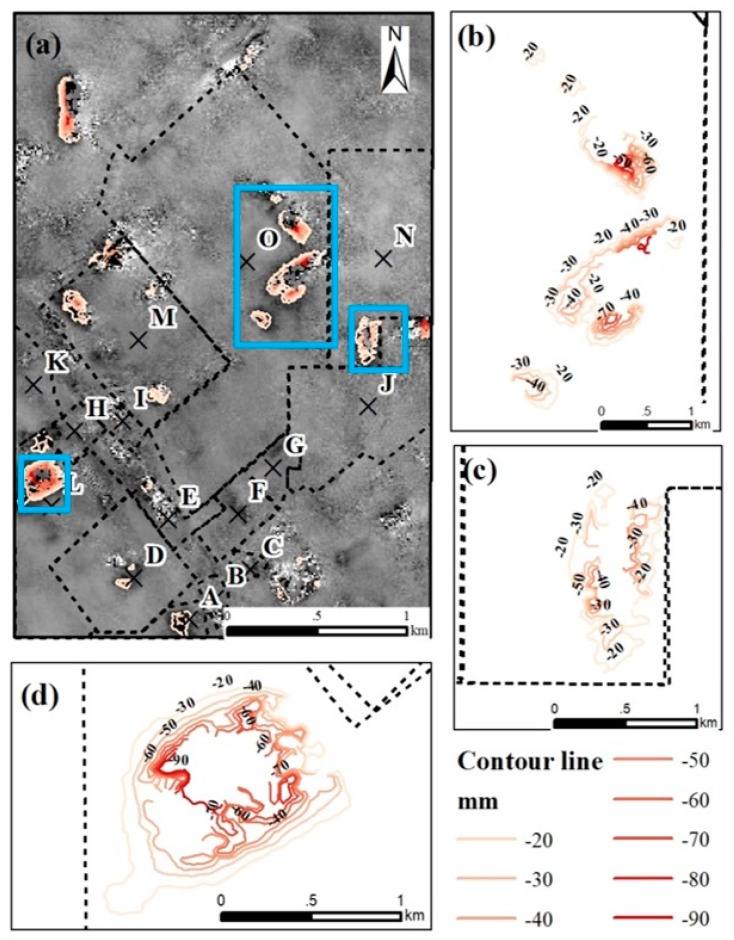
(**a**) Map of the deformation contours obtained from the cumulative displacement from December 2014 to July 2015; (**b**–**d**) the local deformation contours in the O, N and L coal mines.

In addition, the deformation contours map from December 2014 to July 2015 (Figure 11) also shows that three severe subsidence areas were all located in the large-scale coal mines area, which were O, N and L coal mine factories. The O coal mine factory which is large-scale factory and operating more than 20 years is still the severe subsidence area. Especially for the N and L coal mine factory, the construction time indicates two coal mines factories are in the early stages of operation. The operation time of N and L coal mine is almost 3 and 6 years until December 2014.

The results indicate that large coal mine factories operating more than 20 years are more likely to induce land subsidence in this region. Additionally, large-scale coal mines in the early stages of operation may cause severe land subsidence.

**Figure 12:**
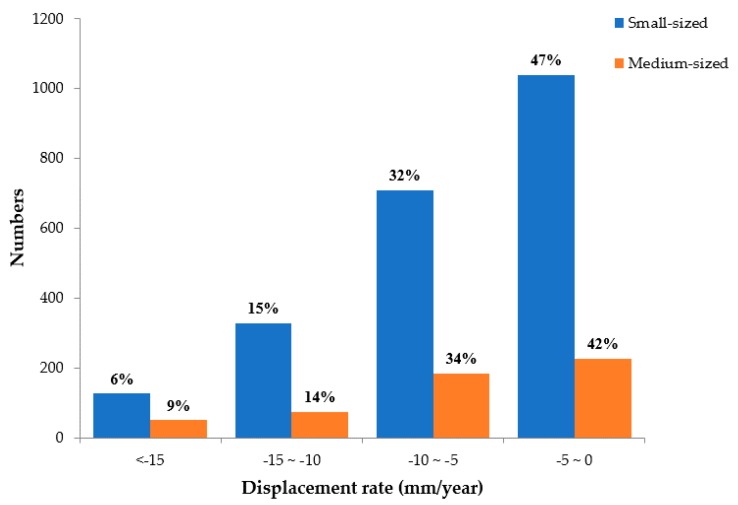
Number of pixels according to 4 classes of displacement rates for small and medium-scale mines estimated by the SBAS-InSAR technique from 2006 to 2011.

To identify the differences in subsidence between small and medium-scale coal mining goafs, the frequency distribution map of the displacement rate from 2006 to 2011 was determined at an interval of 5 mm/year, as shown in Figure 12. In terms of the land displacement range, the range of −5 to 0 mm/year is the main subsidence range for small and medium-scale coal mining, with a slightly higher proportion in small coal mining goafs (47%) than in medium-scale coal mining goafs (42%). According to the statistical results of small and medium-scale coal mines goafs present in Table 4, the effects of the coal mining on land subsidence are small because the maximum settlement rate differs by less than 5 mm/year. 

This finding also demonstrates that the effects of different production volumes between the small and medium-scale coal mines were less than those of large coal mines. 

**Figure 13:**
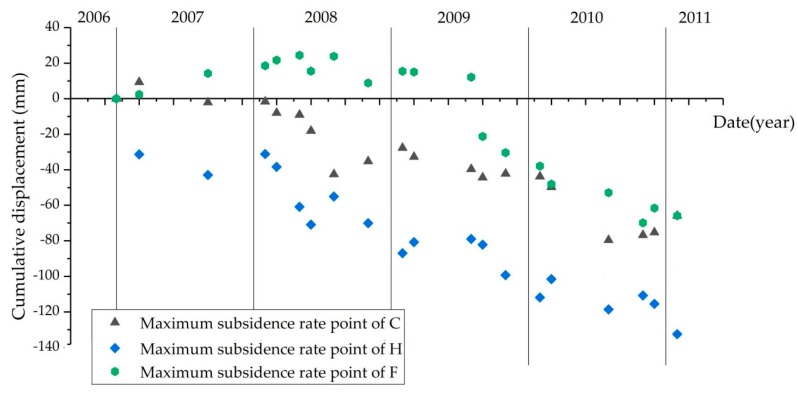
Cumulative displacement for small and medium-scale mines estimated by SBAS-InSAR technique from 2006 to 2011.

Moreover, we select F medium-sized, C small-sized and H small-sized coal mines, which are operating factories until 2012, to perform further analysis on the study of cumulative displacement. The cumulative displacement from December 2006 to January 2011 of the three coal mine factories is shown in Figure 13. 

In Table 2, F medium-sized coal mine is the oldest coal mine, constructed in 1965. The H small-sized coal mine is the most recent coal mines and was constructed in 2006, and the C small-sized coal mine which was established in 2000, is in the early stages of operation. The cumulative settlement of the H coal mine in January 2011 is −132 mm, which is the largest one among the three coal mines. The trend of land subsidence in the H coal mine shows an acute downtrend from 2006 to 2011, while, the surface uplift in the F coal mine occurred from November 2006 to August 2009, and the maximum uplift is 20 mm. Then, the surface begins to subside and reach the value of −66 mm, which is far below that of H coal mine in January 2011.

Therefore, for the small and medium-sized coal mines, it can be concluded that the surface of newly established coal mine factory shows rapid deformation, and the surface of the old mining areas first uplifts and then continues to subside. The reason behind this finding may be the effect of residual subsidence from coal mining and groundwater level changes to meet the needs of underground coal mining [24]. 

Thus, land subsidence caused by large coal mines was inconsistent with that caused by small and medium-scale mines during the period from 2006 to 2011. The subsidence that occurs in large coal mines which have been operating more than 20 years is the most serious in this study area. That is, the effect of the coal mine scale on land subsidence is mainly reflected by a large production volume in the coal mines, exceeding 1.2 million tons per year. Also, the effects of different production volumes between the small and medium-scale coal mines were less than those of the large coal mines. For the small and medium-scale coal mines, the land subsidence caused by new coal mine factories is relatively serious.

## 6. Conclusions

In total, 20 ALOS PALSAR-1 images from December 2006 to January 2011 and 2 ALOS PALSAR-2 images from December 2014 to July 2015 were utilized to investigate land subsidence around coal mining areas through the SBAS-InSAR and D-InSAR techniques in Ordos, Inner Mongolia.

Two periods of land subsidence were obtained by InSAR interpretation and the dangerous risk regions from 2014 to 2015 were deduced, according to the subsidence gradient map from 2006 to 2011. The distance between 50 m to 150 m around the coal mining goafs was regarded as the most dangerous region. Based on these results and combined with information on the coal mines in this study area, large-scale mines with production volumes of more than 1.2 million tons per year, and with operation times of more than 20 years had the greatest effect on land subsidence, while the recently established small-sized and medium-sized coal mines also showed high land subsidence.

There still have possible biases that may degrade the results of this experiment, such as the analysis of InSAR results from multi satellite-based radar platforms. Land subsidence values are obtained along the LOS direction in ALOS-1 and ALOS-2 images. In this mining area, horizontal deformation cannot be neglected. Due to the lack of the simultaneous multi-platform SAR images, we cannot obtain the vertical and horizontal deformation, so in this article, we use the subsidence value along the LOS direction to study the features of land subsidence and the collection of multi-platform SAR data to interpret three-dimensional deformation in the mining area, in order to better deduce the large horizontal movement of the mining area in a small area [44], and verify the dangerous risk regions around the boundary of mining goafs will be a topic of possible future research.

## Figures and Tables

**Table 1:** ALOS PALSAR and ALOS-2 PALSAR-2 information. HH+HV represents dual polarization, FBD represents fine beam dual polarization mode.

Satellite/Sensor	Operation Time	Polarization	Heading	Mode	Number of Acquisitions	Time Spans of Acquisitions
ALOS-1 PALSAR	January 2006–April 2011	HH+HV	Ascending	FBD	20	22 December 2006–2 January 2011
ALOS-2 PALSAR-2	May 2014–Present	HH+HV	Ascending	FBD	2	3 December 2014–15 July 2015

**Table 2:** Coal mining information collect in 2012.

Coal Mine Factory	Scale	Mining Capacity ${(10}^{5}\;tons\;)$	Goafs Area $\left(km^{2}\right)$	Construction Time (year)	Operational Status in 2012
A	Large	30	1.14	1989	Open
B	Medium	6	0	2000	Close
C	Small	3	0.20	2000	Open
D	Large	24	4.54	1988	Open
E	Medium	6	0.09	1984	Close
F	Medium	4.5	0.46	1965	Open
G	Small	3	1.53	1994	Close
H	Small	3	1.29	2006	Open
I	Medium	6	0.28	1993	Close
J	Large	15	1.38	1998	Open
K	Large	27	0.92	1998	Open
L	Large	200	0	2008	Open
M	Large	30	5.10	1988	Open
N	Large	12	0	2011	Construction period
O	Large	50	14.02	1988	Open

**Table 3:** Change rate of high-subsidence gradient areas between two adjacent buffer zones.

**Range**	**50–100**	**100–150**	**150–200**	**200–250**	**250–300**	**300–350**
Change rate	3.94	3.28	2.94	2.20	2.10	1.90
**Range**	**350–400**	**400–450**	**450–500**	**500–550**	**550–600**	**600–650**
Change rate	1.12	1.02	0.58	0.92	0.88	0.82

**Table 4:** Statistics of three scales of coal mining goafs from 2006 to 2011.

Scale	Small	Medium	Large
Number of measurement pixels in the goafs (per ${\mathrm{km}}^{2}$)	1465	1306	1074
Number of measurement pixels with subsidence rates greater than −10 mm/year (per ${\mathrm{km}}^{2}$)	151	149	190
Proportion of subsidence rates (greater than −10 mm/year)	10	11	18
Maximum subsidence rate (mm/year)	−26.69	−22.03	−58.92
